# Role of Nrf2, HO-1 and GSH in Neuroblastoma Cell Resistance to Bortezomib

**DOI:** 10.1371/journal.pone.0152465

**Published:** 2016-03-29

**Authors:** A. L. Furfaro, S. Piras, C. Domenicotti, D. Fenoglio, A. De Luigi, M. Salmona, L. Moretta, U. M. Marinari, M. A. Pronzato, N. Traverso, M. Nitti

**Affiliations:** 1 Giannina Gaslini Institute, Via Gerolamo Gaslini 5, 16147, Genova, Italy; 2 Department of Experimental Medicine, University of Genoa, Via L.B. Alberti 2, 16132, Genova, Italy; 3 Center of Excellence for Biomedical Research, Department of Internal Medicine, University of Genoa, 16132, Genova, Italy; 4 IRCCS-Istituto di Ricerche Farmacologiche “Mario Negri”, Via Giuseppe La Masa 19, 20156, Milano, Italy; 5 Bambino Gesù Children's Hospital, IRCCS, Piazza S. Onofrio 4, 00165, Roma, Italy; II Università di Napoli, ITALY

## Abstract

The activation of Nrf2 has been demonstrated to play a crucial role in cancer cell resistance to different anticancer therapies. The inhibition of proteasome activity has been proposed as a chemosensitizing therapy but the activation of Nrf2 could reduce its efficacy. Using the highly chemoresistant neuroblastoma cells HTLA-230, here we show that the strong reduction in proteasome activity, obtained by using low concentration of bortezomib (BTZ, 2.5 nM), fails in reducing cell viability. BTZ treatment favours the binding of Nrf2 to the ARE sequences in the promoter regions of target genes such as heme oxygenase 1 (HO-1), the modulatory subunit of γ-glutamylcysteine ligase (GCLM) and the transporter for cysteine (x-CT), enabling their transcription. GSH level is also increased after BTZ treatment. The up-regulation of Nrf2 target genes is responsible for cell resistance since HO-1 silencing and GSH depletion synergistically decrease BTZ-treated cell viability. Moreover, cell exposure to all-*trans*-Retinoic acid (ATRA, 3 μM) reduces the binding of Nrf2 to the ARE sequences, decreases HO-1 induction and lowers GSH level increasing the efficacy of bortezomib. These data suggest the role of Nrf2, HO-1 and GSH as molecular targets to improve the efficacy of low doses of bortezomib in the treatment of malignant neuroblastoma.

## 1. Introduction

Cell ability to adapt to oxidative stress by detoxifying reactive oxygen species (ROS) and toxic molecules has been demonstrated to play a role in the failure of different anticancer therapies [1, 2]. Indeed, the imbalance of redox state, induced by different antitumour drugs, can be counteracted by the up-regulation of antioxidants, leading to tumour cell survival. Glutathione (GSH) is generally considered as a leading player among the antioxidant defences and its involvement in cancer cell survival has been widely shown, as reviewed in [3, 4]. However, a central role of heme oxygenase 1 (HO-1) has been demonstrated in tumour progression [5] and the ability to counteract oxidative stress has been related to the antioxidant and antiapoptotic properties of its metabolic products bilirubin [6], ferritin [7] and carbon monoxide [6, 8].

The nuclear factor erythroid 2-related factor 2 (Nrf2) is a transcription factor primarly involved in cell adaption to stress [9]. Toxic molecules and oxidative insults can activate Nrf2 inducing the transcription of a plethora of antioxidant and detoxifying genes [10], among which there are HO-1, both the modifier (GCLM) and the catalytic (GCLC) subunits of γ- glutamylcysteine ligase (GCL), the first rate-limiting enzyme in the synthesis of GSH, and the cysteine/glutamate transporter x-CT [11].

Nrf2, in a resting state, is bound to Kelch-like ECH-associated protein 1 (Keap-1) [12] which acts as adapter protein for the Cullin3 (CUL3) E3 ubiquitin ligase complex and targets Nrf2 for ubiquitination and degradation by proteasome [13]. Oxidative and/or electrophylic stimuli as well as specific Nrf2 phosphorylation enable Nrf2 dissociation from the complex, its nuclear translocation and the activation of specific target genes [14]. Thus, proteasome activity is crucial in the post-translational regulation of Nrf2 and its inhibition can increase Nrf2 level and activity [15].

The inhibition of proteasome activity increases the apoptotic rate, has antitumour efficacy *in vivo* and sensitizes malignant cells to the effects of conventional chemo- and radiotherapies [16–18]. Bortezomib (BTZ) is the first selective and reversible 26S proteasome inhibitor tested in clinical trials [19]. It was approved in 2004 from the U.S. FDA for the treatment of Multiple Myeloma [20] showing impressive results as a single chemotherapeutic agent [20]. Afterwards, it has been proposed also for the treatment of solid tumours, e.g. lung [21, 22]) and renal tumours [23], showing efficacy mainly in combined therapies, as a chemosensitizer [24, 25].

Neuroblastoma (NB) is the most common extracranial solid cancer in childhood. It is extremely heterogeneous, from a low-risk disease, characterized by a good outcome spontaneously or with surgery only, to a high-risk disease, characterized by a high degree of therapeutic failure [26].

The application of bortezomib to the treatment of NB has been proposed [27, 28] but the molecular mechanisms involved in tumour cell response to proteasome inhibition are still largely unknown. Moreover, dose-limiting side-effects such as hepatic and renal toxicity, peripheral neuropathy and myelodysplastic consequences have been described during BTZ-based therapy [29, 30] and impair NB treatment as well [31]. Our recent studies defined the up-regulation of Nrf2 and HO-1 as a molecular mechanism limiting the efficacy of bortezomib in the treatment of highly aggressive NB cells [32].

In the present work, decreasing the dose of BTZ used, we figured out a fine tuning of HO-1 and GSH which synergistically cooperate favouring NB cell resistance to proteasome inhibition. Therefore, we hypothesize that inhibiting HO-1 and depleting GSH can be usefully combined to BTZ therapy and also suggest a possible application of all-*trans-*Retinoic acid (ATRA) in order to inhibit Nrf2-dependent pro-surviving response and improve the efficacy of low doses of bortezomib, increasing the chance of a clinical application.

## 2. Materials and methods

### 2.1 Cell culture and treatments

MYCN amplified, stage IV, HTLA-230 human neuroblastoma cells, obtained from Prof. V. Pistoia (G. Gaslini Institute, Genoa, Italy) [33, 34] were maintained in RPMI 1640 medium (Euroclone, Milan, Italy) supplemented with 10% FBS (Euroclone), 2mM glutamine (Sigma-Aldrich, Milan, Italy), 1% penicillin/streptomycin (Sigma-Aldrich) and 1% amphotericin B (Sigma-Aldrich), 1% Na piruvate (Sigma-Aldrich), 2% amino acids solution (Sigma-Aldrich) at 37°C in a 5% CO_2_ humid atmosphere and sub-cultured every 4 days at 1:5.

Cells were treated for 1–24 h with 2.5 nM and 10 nM bortezomib (Santa Cruz Biotechnology, Santa Cruz, CA, USA). Some samples were co-treated with 3 μM all*-trans*-Retinoic acid (ATRA) or with 1mM buthionine sulfoximine (BSO) (Sigma-Aldrich).

### 2.2 Proteasome activity assay

The activity of proteasome was measured in intact living cells by using the fluorogenic peptide TAT-EDANS-DABCYL (TED) [35]. TED has a proteasome-specific cleavage motif (Leu-Leu-Val-Tyr) fused to TAT and linked to the fluorophores DABCYL and EDANS. TED diffuses freely into the core of proteasome and is specifically recognized and hydrolyzed, in a ubiquitination-independent way, by the 20S proteasome chymotrypsin-like activity, generating a fluorescent reporter of proteasome activity *in vivo*. Cells were seeded in 96 well plates and treated for 1–24 h with 2.5 nM or 10 nM bortezomib. At the end of incubation, after 3 washes with Hanks' balanced salt solution (HBSS), cells were incubated with HBSS containing 10 μM TED. The EDANS-dependent fluorescent signal (excitation 340 nm, emission 510 nm) was monitored with a microplate reader (Infinite F500, Tecan, Männedorf, Switzerland) up to 60 minutes. The results were expressed as arbitrary units of fluorescence.

### 2.3 Small interfering RNA

Small interfering RNA was performed by using a specific pool of oligonucleotides against human HO-1 (On-TargetPlus SMART pool human heme oxygenase 1; Dharmacon, Lafayette, CO, USA), as previously described [36]. Briefly, cells were transfected with 10–50 nM HO-1 siRNA (siHO-1) for 24 h to set up the best experimental condition, by using Polyplus-Transfection Interferin (Euroclone), according to the manufacturer's instructions. The negative control was performed by transfection with a non targeting silencing pool (On-TargetPlus siControl nontargeting pool; Dharmacon) at the same concentration used for siHO-1. The efficiency of silencing was evaluated by RT-PCR. 50 nM siHO-1 for 24h was used for the next experiments and cells were incubated with siHO-1 contextually with 2.5 nM BTZ.

### 2.4 Dominant negative Nrf2 over-expression

HTLA-230 cells were transiently transfected with pmaxFPTM-Green-C empty vector (pmax empty vector) or pmaxFPTM-Green-C containing the dominant negative Nrf2 protein (pmax Nrf2-DN), kindly provided by Prof. M. Ciriolo [37], by using Lipofectamine 2000 (Life Technologies, Monza, Italy) according to the manufacturer’s instructions. Transfection efficiency was checked after 48 h by fluorescence microscopy. After transfection, cells were exposed to 2.5 nM BTZ for further 24 h. The efficacy of Nrf2-DN over-expression in inhibiting Nrf2-dependent pathways was confirmed by measuring the mRNA expression and protein levels of Nrf2 target genes.

### 2.5 Cytotoxicity assay

The cytotoxicity was evaluated by counting Trypan blue dye-stained cells. Briefly, HTLA-230 cells seeded in 6-well plates were treated as previously described. At the end of the treatments cells were detached with trypsin, stained with Trypan blue dye, and counted microscopically. MTT assay was also used, as previously reported [32].

### 2.6 Evaluation of glutathione content

Evaluation of total GSH content (tGSH) was performed by HPLC according to Fariss and Reed [38], as already described in [39]). tGSH was evaluated in nEq by the analysis of the chromatograms, calculated as GSH+2GSSG, and normalized on protein content [40]. Evaluation of GSSG was performed by Asensi’s method [39, 41], which requires rapid blockage of –SH group by N-ethylmaleimide, in order to avoid in vitro oxidation of the reduced form of glutathione. Also GSSG was evaluated in nEq/mg of protein.

### 2.7 RNA extraction and RT-PCR

Total RNA was extracted using TRIZOL reagent (Life Technologies) according to the manufacturer’s instructions and was then reverse transcribed into cDNA by random hexamer primers and SuperScriptTM II Reverse Transcriptase (Life Technologies). Amplification of cDNA by polymerase chain reaction was performed using Platinum Taq DNA Polymerase (Life Technologies) and specific primers for human proteasome subunit β1 (PSBM6), β2 (PSBM7), β5 (PSBM5), HO-1, GCLM, x-CT and GCLC. Ribosomal 18S expression was used as the housekeeping gene. Primer sequences used (Tib Mol Biol, Genoa, Italy) were: proteasome subunit β1, PSMB6 Fw 5' tcc ggg agc tcc tac atc tat g 3'; PSMB6 Rv 5' ctc tgc aat ggc tgc cag gc 3' (156 bp); proteasome subunit β2, PSMB7 Fw 5' agg gat ggt tgt tgc tga ca 3'; PSMB7 Rv 5' aac tag ggc tgc acc aat gt 3'(232 bp); proteasome subunit β5, PSMB5 Fw 5' gtt ccg cca tgg agt cat agt 3'; PSMB5 Rv 5' ccg gtt ccc ttc act gtc cac 3' (340 bp); HO-1 Fw 5’ gtc caa cat cca gct ctt tga gg 3’; HO-1 Rv 5’ gac aaa gtt cat ggc cct ggg a 3’ (284 bp); GCLM Fw 5’ cca gat gtc ttg gaa tgc 3’; GCLM Rv 5’ tgc agt caa atc tgg tgg 3’ (408 bp); x-CT Fw 5’ cgt cct ttc aag gtg cca ctg 3’; x-CT Rv 5’ tgt ctc ccc ttg ggc aga ttg 3’ (295 bp); GCLC Fw 5’ atg gag gtg caa tta aca gac 3’; GCLC Rv 5’ act gca ttg cca cct ttg ca 3’ (206 bp); 18s Fw 5’ ggg gcc cga agc gtt tac t 3’; 18s Rv 5’ ggt cgg aac tac gac ggt atc 3’ (296 bp). PCR products were separated by electrophoresis on 2% agarose gel pre-stained with ethidium bromide, visualized under UV light and quantified by densitometric analysis by using a specific software (GelDoc, BIO-RAD, Milan, Italy).

### 2.8 Western Blotting

After the treatments, total protein extraction was performing using RIPA buffer [50 mM Trizma hydrochloride, pH 7.4 (Sigma Aldrich), 150 mM NaCl (Carlo Erba Reagents, Italy), 1% Igepal CA-630 (Sigma Aldrich), 0.1% Sodium dodecyl sulfate (Sigma Aldrich), supplemented with 1 mM Phenylmethanesulfonylfluoride (Sigma Aldrich), 1% Phosphatase Inhibitor Cocktail 3 (Sigma Aldrich) and with Protease Inhibitors (Roche Diagnostics, Milan, Italy)] as previously described [36]. Protein content was determined using the bicinchoninic acid assay (BCA,Pierce,ThermoScientific, Rockford, USA). Lysates were subjected to gel electrophoresis using Mini-Protean TGX ^™^ Gels precast (BIO-RAD, Milan, Italy) and immunoblotted with antibodies against HO-1 (rabbit polyclonal antibody, ORIGENE, Herford,Germany), GCLM (rabbit polyclonal antibody generously provided by Dr. T.J. Kavanagh, University of Washington, Seattle, USA) [42, 43], x-CT /SLC7A11 (rabbit mAb, Cell Signaling, Danvers, MA, USA) and Tubulin (mouse antibody, abcam, Cambridge, UK). Enhanced chemiluminescence was used to visualize bands on autoradiographic films (GE Healthcare, Buckinghamshire, UK) and quantified by using Gel Doc 2000 densitometer (BIO-RAD).

### 2.9 Chromatin immunoprecipitation assay

Nrf2 binding to ARE sequences in the promoter regions of HO-1, GCLM and x-CT was assessed by chromatin immunoprecipitation (ChIP) [44]. HTLA-230 cells were grown in T75 flasks and treated with 2.5 nM BTZ, 3 μM ATRA and their association (2.5 nM BTZ + 3 μM ATRA) for 6 and 24 hours. Following treatment, cells were crosslinked with 1% formaldehyde at 37°C for 10 min and then sonicated. Supernatants were immunocleared with Salmon Sperm DNA/protein A agarose (Merk Millipore, Milan, Italy) and immunoprecipitated with anti Nrf2 C-20 (Santa Cruz) or with Normal Rabbit IgG (Merk Millipore). An aliquot of the precleared chromatin was used as the input DNA. DNA extraction was perform by using UltraPure Phenol:Chloroform:Isoamyl Alcohol (25:24:1) (Life Technologies). 1 μl of DNA immunoprecipitated and 1 μl of input DNA were amplified by PCR using Platinum Taq DNA Polymerase (Life Technologies) and specific primers for the promoter regions of HO-1 (Enhancer E1 and E2) [45], GCLM [46] and x-CT [47]. The amplification products were analyzed in a 2% agarose gel stained with ethidium bromide, visualized under UV light and quantified by densitometric analysis by using a specific software (GelDoc, BIO-RAD). All values were normalized to input DNA.

Primers set used were: HO-1 E1 Fw 5' gct gcc caa acc act tct gt 3'; HO-1 E1 Rv 5' gcc ctt tca cct ccc acc ta 3'; HO-1 E2 Fw 5' tcc ttt ccc gag cca cgt g 3'; HO-1 E2 Rv 5' tcc gga ctt tgc ccc agg 3'; GCLM ARE Fw 5' cgc ggg atg agt aac ggt 3'; GCLM ARE Rv 5' gag agc tga ttc caa act g 3'; x-CT ARE Fw 5' gct tag gtc agt tga gca a 3'; x-CT Rv 5' cat tac aca cca gct cag ct 3'.

### 2.10 Statistical analysis

The data are presented as a mean ± standard error from at least three independent experiments. As far as the RT-PCR and Western Blotting analysis is concerned, the results are expressed as relative amounts with respect to the control. However, since the expression of HO-1 in control cells is almost under the limit of detection, in order to perform quantification and statistical analysis, the expression of HO-1 in BTZ-treated cells has been considered as 100%. The statistical significance was evaluated by one-way ANOVA followed by Dunnett's post-test, or by t-test to compare two groups, using GraphPad Prism software (San Diego, CA, USA).

## 3. Results

### 3.1 A low dose of bortezomib (BTZ, 2.5 nM) strongly decreased proteasome activity, did not change viability and did not modify the transcription of proteasome subunits in high-risk NB cells

**Fig 1:**
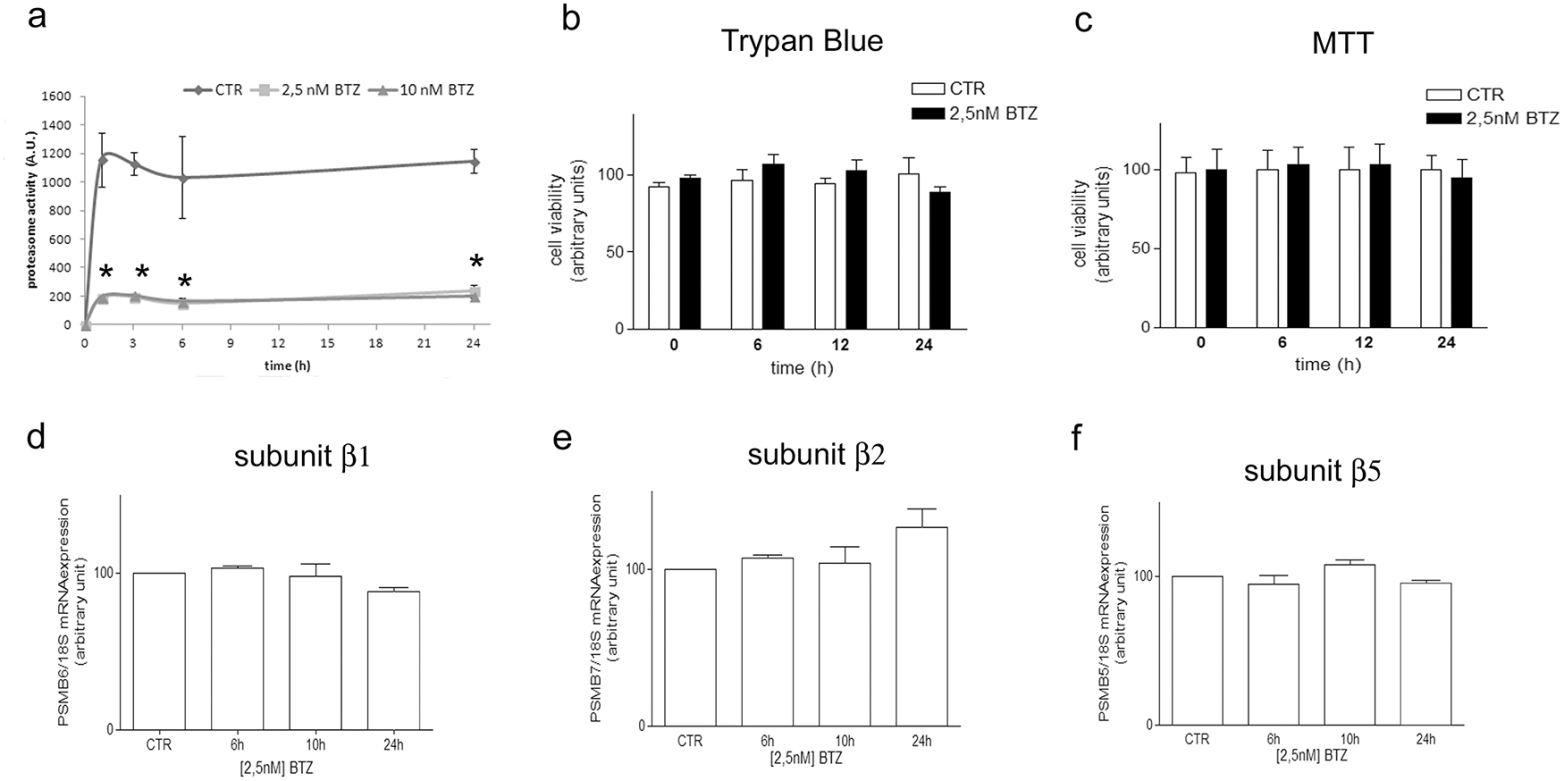
A low dose of BTZ decreases proteasome activity with no effect on cell viability. HTLA-230 NB cells were treated with 2.5 nM BTZ for 1–24 h. The chymotrypsin-like protease activity was measured in living cells by using the fluorescent probe TED. The efficacy of 2.5 nM BTZ in inhibiting proteasome activity was compared with the effect induced by a higher dose of BTZ (10 nM) (a). The number of viable cells were detected by Trypan blue dye exclusion test (b) and MTT assay (c). mRNA level of proteasome subunit β1 (d), subunit β2 (e), subunit β5 (f) were measured by means of RT-PCR. Total RNA was extracted from HTLA-230 cells treated with 2.5 nM BTZ up to 24 h, as indicated. PCR products were separated by electrophoresis and the relative intensities of the bands were normalized to 18S expression. The graphs show the mean value of three independent experiments (mean±SE); *p<0.01 vs untreated cells for both 10 and 2.5 nM BTZ.

HTLA-230 cell exposure to 2.5 nM BTZ strongly reduced proteasome activity. Indeed, the chymotrypsin-like protease activity was reduced of 80% already after 1 h, up to 24 h and no further decreases in proteasome activity were detected using 10 nM BTZ (Fig 1a). However, cell viability was not modified by the exposure to 2.5 nM BTZ, as shown by both Trypan blue exclusion test (Fig 1b) and MTT assay (Fig 1c). The inefficacy of BTZ in modifying HTLA-230 viability has been confirmed also at 10 nM concentration, as already reported in a previous study [32]. Moreover, 24 h exposure to 2.5 nM BTZ did not modify mRNA expression of the proteolytic core subunits β1 (PSMB6), β2 (PSMB7) and β5 (PSMB5) (Fig 1d, 1e and 1f respectively).

### 3.2 Bortezomib treatment up-regulated HO-1 expression and increased GSH levels

**Fig 2:**
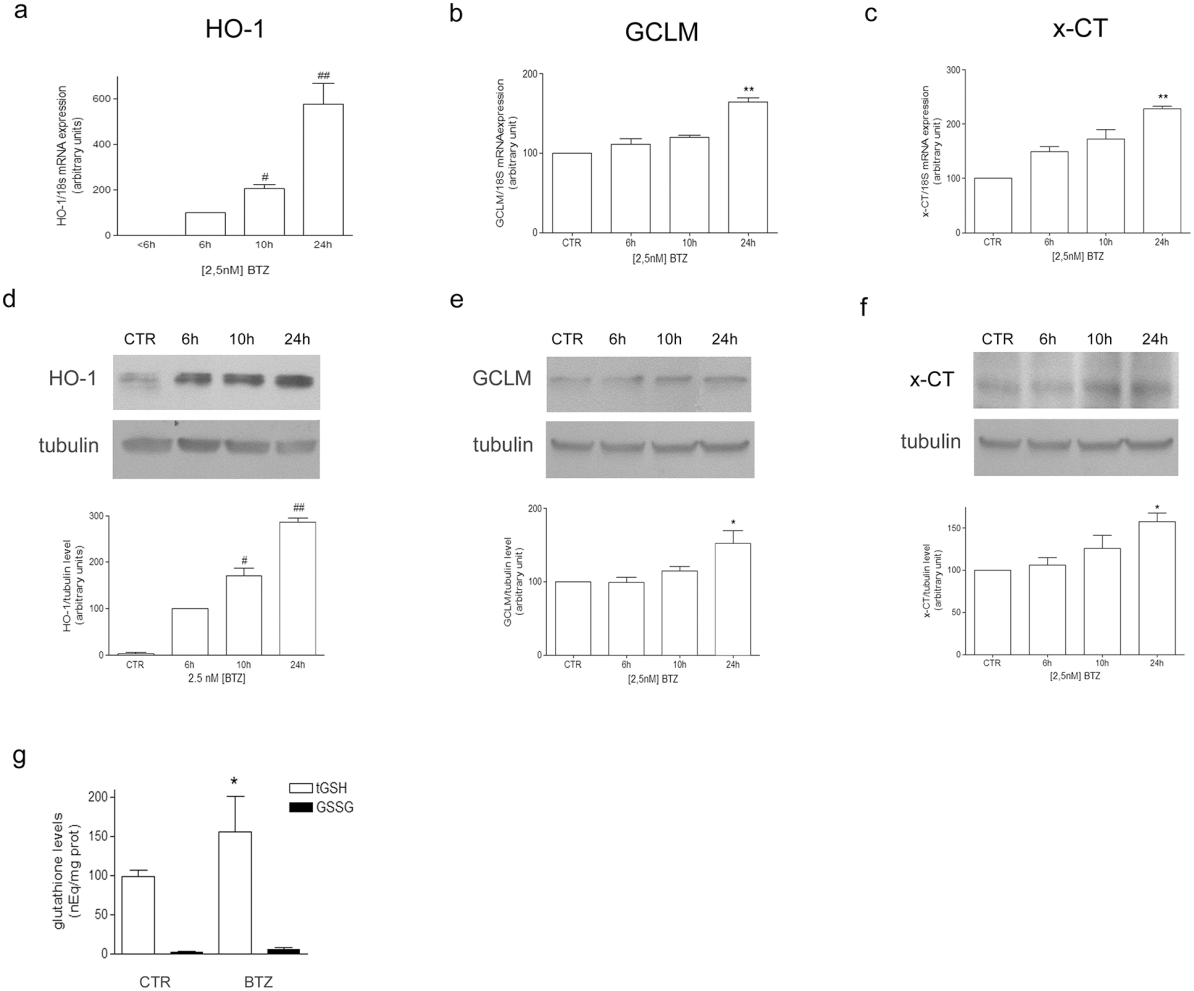
BTZ enhances mRNA expression of HO-1, GCLM and x-CT and increases total GSH level. mRNA and protein level of HO-1 (a and d, respectively), GCLM (b and e, respectively) and x-CT (c and f, respectively) were measured by means of RT-PCR and Western Blotting in cell treated with 2.5 nM BTZ up to 24 h, as detailed in Materials and Methods. PCR products were separated by agarose electrophoresis and the relative intensities of the bands were normalized to 18S expression (a, b, c). The intensities of protein bands were normalized to the level of tubulin (d, e, f). The bar graphs show the mean values of three independent experiments (mean±SE). The bands show one representative experiment. In (g) tGSH and GSSG content was measured by HPLC in cells treated with 2.5 nM BTZ for 24 h. The graph shows the mean values of three independent experiments (mean±SE). In (a and d) #p<0.05 and ##p<0.01 vs 6 h BTZ-treated cells. In (b, c, d, e and g) *p<0.05 and **p<0.01 vs untreated cells.

In order to show the involvement of Nrf2-dependent antioxidant responses in cell resistance to BTZ, the mRNA expression and the protein levels of heme oxygenase 1 (HO-1), γ-glutamylcysteine ligase (both modulatory GCLM, and catalytic GCLC subunits) and the transporter for cysteine (x-CT) have been measured. HO-1 mRNA was detectable only after 6 h of BTZ treatment and progressively increased up to five fold at the longest experimental time (Fig 2a). The mRNA level of GCLM (Fig 2b) and x-CT (Fig 2c) significantly increased by 64% and 105% respectively only after 24 h. No changes of GCLC were detected (data not shown). Accordingly, protein level of HO-1 was clearly detectable after 6h of BTZ treatment, increasing up to three fold after 24h in comparison to the value at 6h (Fig 2d). Moreover, after 24h of BTZ treatment GCLM and x-CT protein levels were increased (+45% and +57% vs untreated cells, respectively. Fig 2e and 2f). No changes were observed at the shortest experimental times (1 and 3h) with regard to all considered genes (data not shown).

In addition, cell exposure to BTZ significantly increased the content of total GSH of about 57 nEq/mg prot in comparison to untreated cells. In the same experimental conditions, the level of GSSG was increased of about 2.5 nEq/mg prot in comparison to control cells, with no statistical significance (Fig 2g).

### 3.3 HO-1 silencing and GSH depletion synergistically increased cell sensitivity to bortezomib

**Fig 3:**
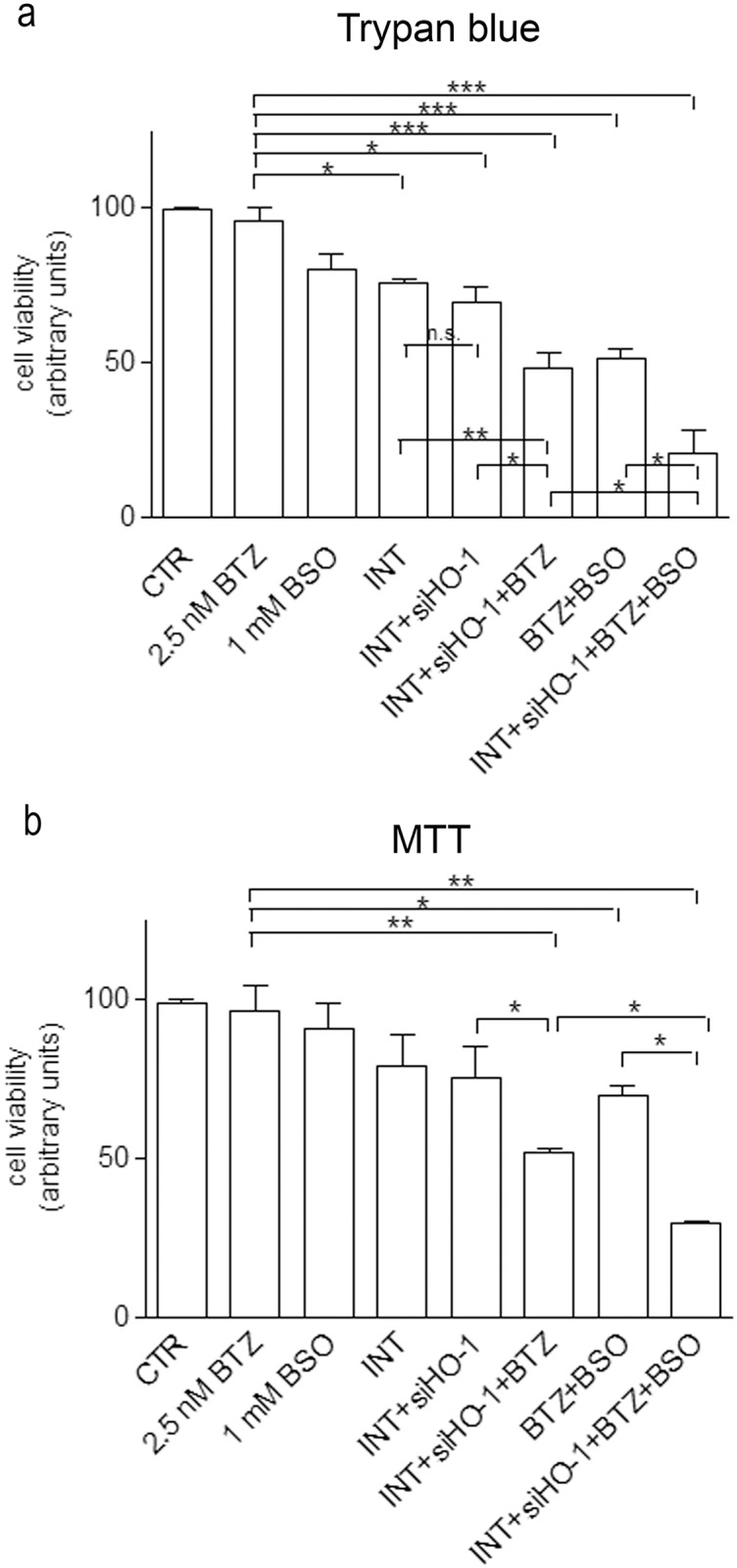
GSH depletion and HO-1 silencing improve the efficacy of BTZ in reducing cell viability. The number of viable cells were detected by Trypan blue dye exclusion test (a) and MTT assay (b). Cells were treated for 24 h with 2.5 nM BTZ, 1 mM BSO or silenced for HO-1, as indicated. Some samples were treated with the siRNA transfection reagent Interferin (INT) alone, as internal control. The graphs show the mean value of three independent experiments (mean±SE); *p<0.05; **p<0.01; ***p<0.001; n.s. no significant.

To prove the involvement of HO-1 and GSH in cell resistance to proteasome inhibition, BTZ-treated cells have been silenced for HO-1 and/or depleted of GSH by using 1 mM BSO and cell viability was measured by Trypan blue dye test (Fig 3a) and MTT assay (Fig 3b). As already shown in Fig 1, BTZ alone did not change cell viability. Moreover, BSO treatment *per se* had a mild, not significant, effect in reducing cell viability while the association of BTZ with BSO significantly reduced cell viability in comparison to BTZ-treated cells (-44% as shown by Trypan blue test and -26.6% as shown by MTT assay).

HO-1 silencing (siHO-1) reduced the viability of untreated cells (-29.8% and -23% vs CTR as shown by Trypan blue and MTT respectively) but the transfection medium (Interferin, INT) *per se* was able to reduce cell viability of 24% and 18% as measured by the two methods. However, siHO-1 significantly decreased BTZ-treated cell viability (-47.4% and -44.5% vs BTZ alone as shown by Trypan blue test and MTT assay). Moreover, the association of HO-1 silencing with BTZ and BSO further reduced cell viability (-75% and -66.8% vs BTZ alone, as shown by Trypan blue and MTT respectively).

The ability of siHO-1 in reducing HO-1 expression and the efficacy of BSO in depleting GSH are shown in the supporting information (S1 and S2 Figs) respectively.

### 3.4 Nrf2 activation enabled the up-regulation of HO-1 and GSH in BTZ-treated cells

**Fig 4:**
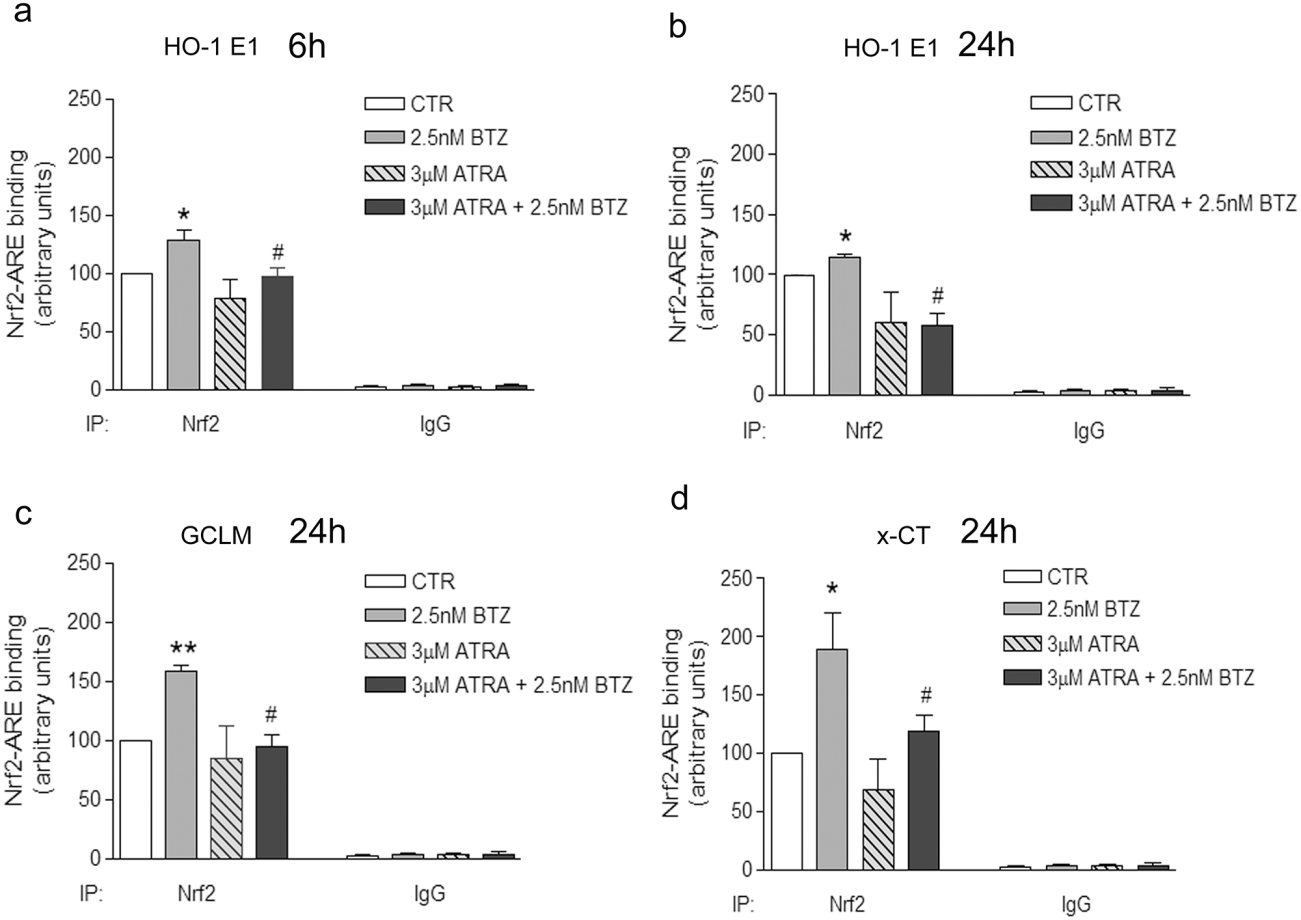
ATRA decreases the binding of Nrf2 to ARE sequences in the promoter regions of HO-1, GCLM and x-CT in BTZ-treated cells. The binding of Nrf2 to ARE was evaluated by means of ChIP in HTLA-230 cells treated with 2.5 nM BTZ, 3 μM ATRA and 3 μM ATRA + 2.5 nM BTZ. Chromatin was immunoprecipitated with anti Nrf2 or with Normal IgG antibody, as indicated. PCRs were done with primers designed for the promoter regions of HO-1 E1 enhancer (in a and b, after 6 h or 24 h of cell treatment respectively), GCLM (in c, after 24 h) and x-CT (in d, after 24 h), as detailed in Material and Methods section. The amplification of pre-cleared DNA (input) has been done to perform the normalization of results. The graphs show the mean value of three independent experiments (mean±SE); *p<0.05 and **p<0.01 vs CTR cells; #p<0.05 vs BTZ-treated cells.

Considering the role of proteasome in the regulation of Nrf2 activity, we checked the ability of Nrf2 to bind ARE sequences in the promoter regions of HO-1, GCLM and x-CT in BTZ-treated cells. HO-1 promoter contains two ARE sequences in the two enhancers E1 and E2 [48]. As shown in Fig 4a, the binding of Nrf2 to E1 was up-regulated by BTZ already after 6 h (+28% vs CTR). No changes were detected at 6 h, as far as HO-1 E2, GCLM and x-CT are concerned (data not shown). Moreover, after 24 h of BTZ treatment, Nrf2-ARE binding significantly increased in HO-1 E1 (+14.6% vs CTR, Fig 4b) and, with even greater extent, in the promoter regions of GCLM (+58.3% vs CTR, Fig 4c) and x-CT (+88.5% vs CTR, Fig 4d), while no changes were observed with regard to HO-1 E2 (data not shown).

In addition, since low doses of all-*trans*-Retinoic acid (ATRA) have been demonstrated to inhibit Nrf2 activity [49], the efficacy of 3 μM ATRA in reducing Nrf2-ARE binding in BTZ-treated cells was tested.

Cell co-treatment with ATRA and BTZ effectively reduced Nrf2-ARE binding in HO-1 E1 already after 6 h (Fig 4a) and, after 24 h, in HO-1 E1, GCLM and x-CT promoters (-40% vs BTZ-treated cells, Fig 4b, 4c and 4d).

**Fig 5:**
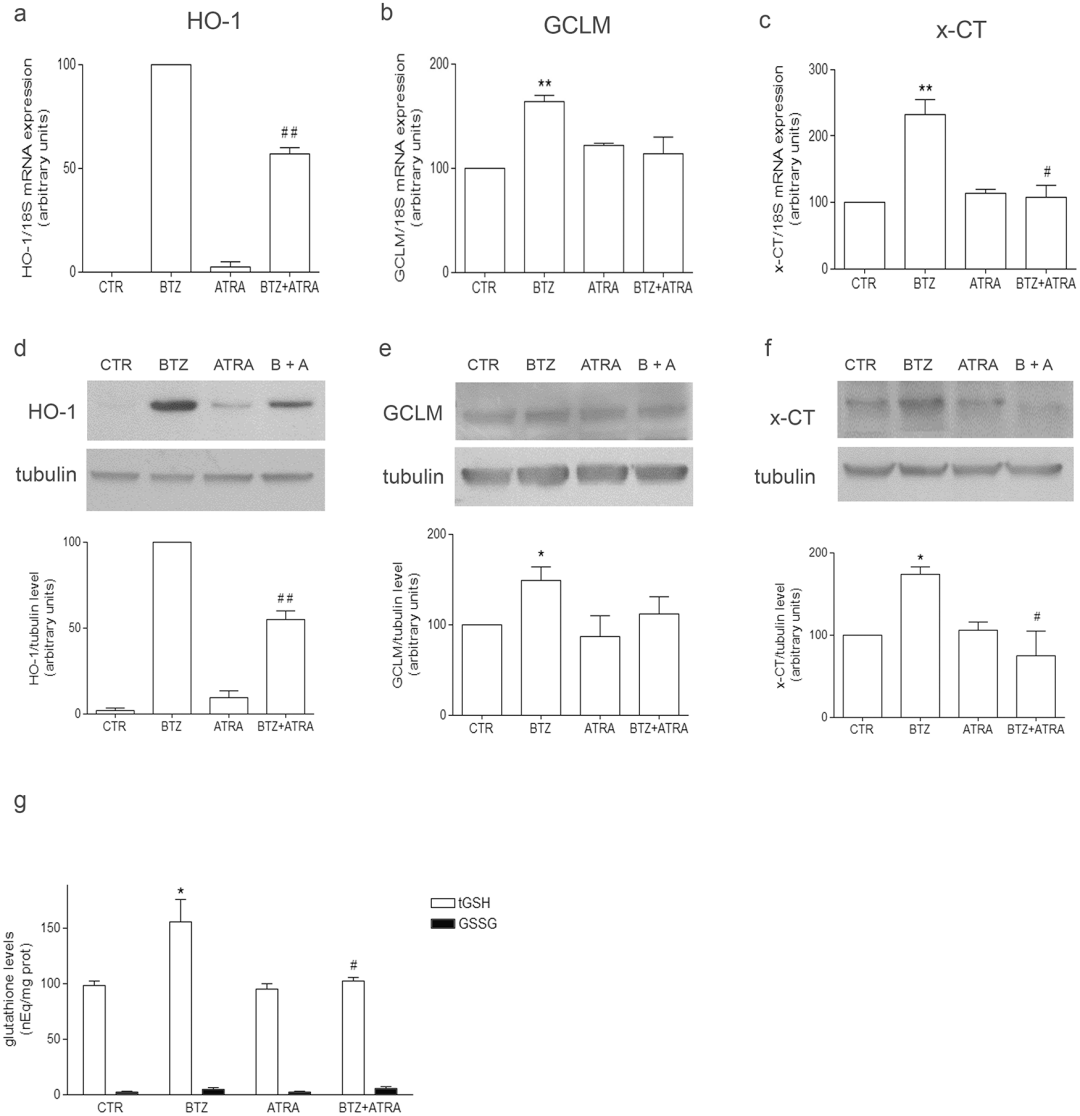
ATRA decreases mRNA expression of HO-1, GCLM and x-CT and lowers GSH level in BTZ-treated cells. mRNA and protein levels of HO-1 (a and d, respectively), GCLM (b and e, respectively) and x-CT (c and f, respectively) were detected by means of RT-PCR and Western Blotting in cells treated with 2.5 nM BTZ, 3 μM ATRA and 3 μM ATRA + 2.5 nM BTZ for 24 h, as detailed in Materials and Methods. The bar graphs show the mean values of three independent experiments (mean±SE). The bands show one representative experiment. In (g) tGSH and GSSG content was measured by HPLC in cells treated with 2.5 nM BTZ and 3 μM ATRA for 24 h, as indicated, and the graph shows the mean values of three independent experiments (mean±SE); * p<0.05 and **p<0.01 vs untreated cells; #p<0.05 and ##p<0.01 vs BTZ-treated cells.

Furthermore, co-treatment with ATRA and BTZ significantly reduced the mRNA expression (-43% vs BTZ, Fig 5a) and the protein level of HO-1 (-46% vs BTZ, Fig 5d) and completely counteracted the up-regulation of x-CT (Fig 5c and 5f). Only a partial effect was observed on the induction of GCLM, in terms of both RNA expression and protein level (Fig 5b and 5e). Moreover, the co-treatment with ATRA and BTZ was able to bring the content of tGSH back to the level of control with no significant changes as far as the level of GSSG is concerned (Fig 5g).

### 3.5 Nrf2-inhibition reduced cell viability in bortezomib-treated cells

**Fig 6:**
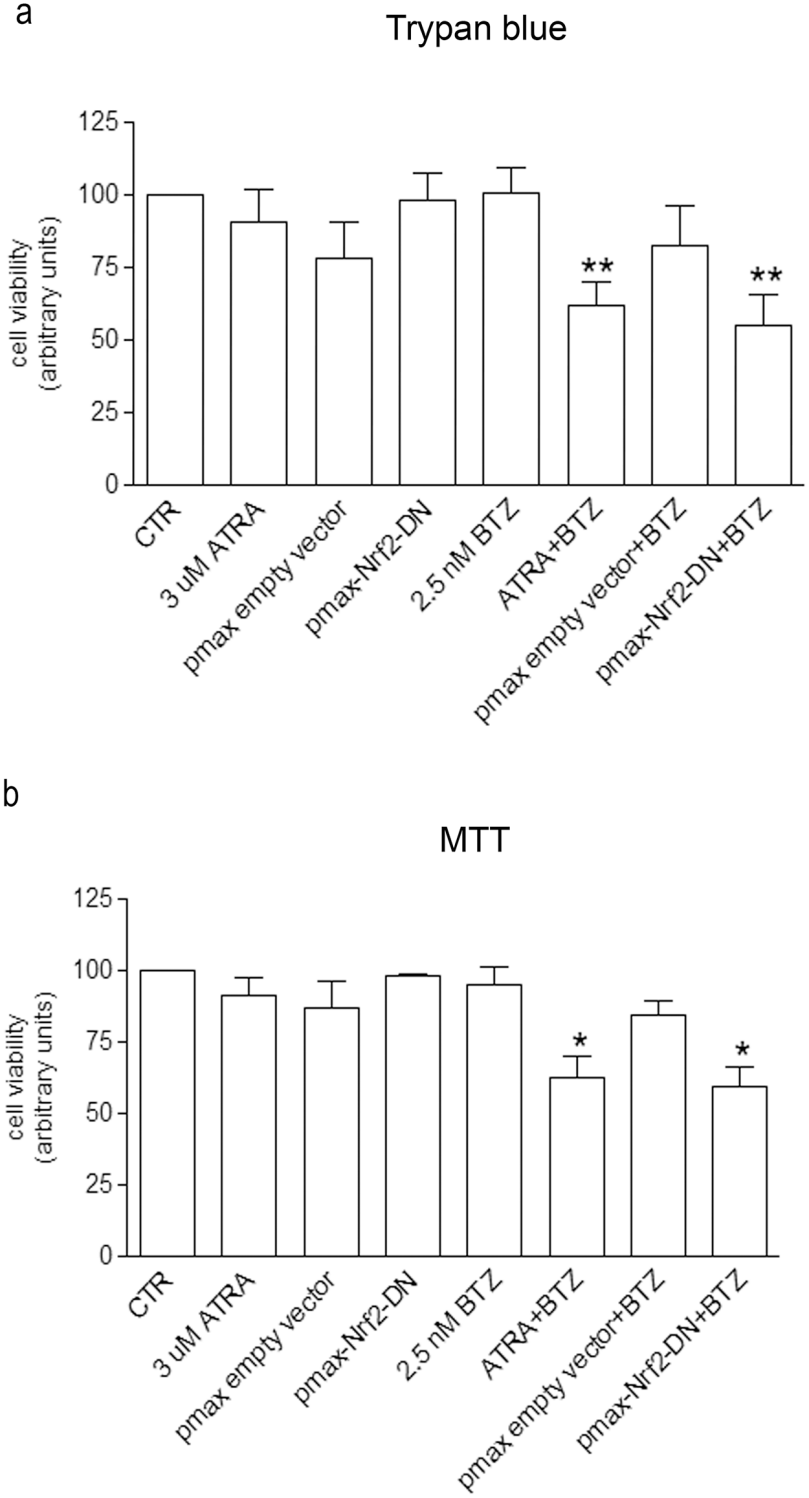
ATRA, as well as the overexpression of a dominant negative form of Nrf2, reduces BTZ-treated cell viability. Cell viability was detected by Trypan blue exclusion test (a) and MTT assay (b). Cells were treated for 24 h with 2.5 nM BTZ and 3 μM ATRA, as indicated. Some samples were transfected with a plasmid coding for a dominant negative form of Nrf2 (pmax-Nrf2-DN) in order to inhibit Nrf2 activity. The internal control of the transfection has been performed using an empty plasmid (pmax-empty vector). The graphs show the mean value of three independent experiments (mean±SE); *p<0.05 and **p<0.01 vs BTZ-treated cells.

To prove the involvement of Nrf2 in cell resistance to BTZ, we checked the viability of BTZ-treated cells in presence of ATRA. Moreover, the viability of cells over-expressing a dominant negative form of Nrf2 (Nrf2-DN) exposed to BTZ was tested. Both Trypan blue dye exclusion test and MTT assay have been performed (Fig 6a and 6b respectively). Co-treatment with ATRA and BTZ significantly reduced cell viability in comparison with cell exposure to BTZ alone (-38% and -32.7% vs BTZ alone in a and b respectively). Similar results were obtained in Nrf2-DN over-expressing cells treated with BTZ (-45% and 35.3% vs BTZ alone in a and b respectively). The efficacy of Nrf2-DN in inhibiting HO-1, GCLM and x-CT expression was tested by RT-PCR and Western Blotting analyses and the results are shown in the supporting information (S3 Fig).

## 4. Discussion

In this work we show the crucial role played by Nrf2, HO-1 and GSH, in the development of chemoresistance in highly aggressive neuroblastoma (NB) cells treated with bortezomib. Antioxidants are known to provide cancer cells with resistance to anticancer therapies which often act by generating oxidative stress [2]. GSH has always been considered to be the main player in counteracting redox unbalance favouring cancer cell survival but, more recently, also other antioxidants such as uric acid [50] or bilirubin [51, 52], have been proved to have a role in the progression of different kinds of tumour.

In the therapy of NB, BTZ has been already proposed, mainly in combination with other chemotherapeutic drugs [27, 28], but, especially in the high-risk disease, the efficacy is limited and tumour resistance is observed. Moreover, toxic side effects are observed at the commonly used doses [31]. In this context, our previous study demonstrated that the up-regulation of HO-1 is a crucial mechanism involved in the resistance of highly aggressive HTLA-230 NB cells to proteasome inhibition induced by 5–40 nM BTZ [32]. Therefore, in the present work NB cells were exposed to a lower dose of BTZ, 2.5 nM, which was able to strongly reduce proteasome activity in a rapid and long-lasting way, with the same efficacy of a higher dose, but failed in reducing the number of viable cells.

It is known that Nrf2 is peculiarly regulated by proteasome activity which is necessary to maintain its low basal level [53] and the inhibition of proteasome activity can increase Nrf2-dependent gene activation [54, 55]. Since Nrf2 is involved in the transcription of proteasome [56–58], the expression of subunits β1, β2 and β5 has been checked in our experimental conditions and no changes have been observed. However, Nrf2 crucially drives cell adaption to oxidative stress by running the transcription of a plethora of antioxidant and detoxifying genes, among which HO-1, GCLM and x-CT. In addition, the role of Nrf2 and its downstream targets has been recently highlighted in cancer cell resistance to therapies [59, 60]. Therefore, we analysed the binding of Nrf2 to ARE sequences in the promoter regions of target genes (HO-1, GCLM and x-CT) in order to assess Nrf2 activity and Nrf2-dependent gene activation. Indeed, due to the complex regulation of Nrf2 level and cytosol-nucleus shuttling [61], the detection of protein level or localization might be meaningless. Thus, by means of chromatin immunoprecipitation analysis, we have shown an increase in the binding of Nrf2 to ARE in BTZ-treated cells. These results are consistent with the ones obtained by RT-PCR and WB analyses confirming that all the same genes are up-regulated by BTZ. The only apparent inconsistency is related to the binding of Nrf2 to ARE in the promoter region of HO-1 in untreated cells. HO-1 is an inducible gene and its mRNA level is almost undetectable in untreated cells. However, ChIP assay shows that Nrf2 is bound to HO-1 promoter also in untreated cells. It has been already demonstrated that the induction of HO-1 transcription is a complex phenomenon, regulated not only by the binding of Nrf2 to ARE but also by the presence of other proteins such as Brahma-related gene 1 (BRG-1), which is an essential cofactor needed for the recruitment of RNA polimerase II [45] and its level is regulated by proteasome activity as well [62]. In our model, HO-1 expression becomes detectable after 6 h and progressively increases up to 24 h of BTZ treatment and, at this time, also the modulatory subunit of γ-glutamylcysteine ligase (GCLM) and the transporter for cysteine (x-CT) are over-expressed. Moreover, the level of tGSH is significantly increased after BTZ treatment without significant alterations of its oxidized form. Thus, the enhancement of tGSH is mainly related to the increase of reduced GSH and is likely to be due to GSH synthesis, even though the level of GCLC is not modified at all. Indeed, it has been demonstrated that the modifier subunit GCLM plays a leading role in regulating the activity of the GCL [63–65]. In addition, the significant up-regulation of x-CT induced by BTZ, through the increased availability of cysteine, could be able to drive the synthesis of GSH, even if the level of GCL was not modified, as recently shown by others in astrocytes [66].

Therefore, we provide evidence that HO-1 and GSH are responsible for cell adaption to proteasome inhibition: indeed, GSH depletion obtained using BSO, as well as HO-1 silencing, reduces the viability of BTZ-treated cells. Furthermore, the association of BTZ with HO-1 silencing and BSO dramatically reduces cell viability, showing a synergistic effect. It is important to note that BSO alone, even though it brings the level of GSH under the limit of detection, causes only a mild decrease of cell viability, confirming that NB cells are able to adapt easily to oxidative stress and that a multi-targeted approach is needed to overcome chemoresistance.

In order to demonstrate the involvement of Nrf2 in the activation of HO-1, GCLM and x-CT, Nrf2 activity in BTZ-treated cells has been inhibited by all-*trans*-Retinoic acid (ATRA). Indeed, retinoic acid receptors RARalpha and RXR have been demonstrated to interfere with the binding of Nrf2 to ARE sequences, decreasing the transcription of its target genes [49, 67]. In the present study, the association of BTZ with ATRA significantly reduces the binding of Nrf2 to ARE in the promoter regions of HO-1, GCLM and x-CT in comparison to cells treated with BTZ alone. As a consequence, the BTZ-induced up-regulation of HO-1 and x-CT is prevented by ATRA co-treatment, and the level of tGSH is brought back to the level of untreated cells, without any significant effect on GSSG. Instead, the effect of the ATRA + BTZ co-treatment on GCLM expression is limited and does not reach statistical significance in comparison to BTZ alone.

Other papers, indeed, have demonstrated the efficacy of ATRA in preventing Nrf2 binding on its target genes [58] and recently the efficacy of ATRA in reducing HO-1 transcription and in decreasing GSH content has been clearly shown in leukemia cells [68]. Moreover, to our knowledge, this is the first evidence about the efficacy of ATRA in reducing x-CT and, though partially, GCLM expression. However, the molecular mechanism that underlies the efficacy of ATRA in reducing GSH content with only a partial effect on GCLM expression deserves more investigation. We can speculate that, in our experimental condition, the enhanced availability of cysteine, related to the expression of its transporter x-CT, could be the driving force in GSH synthesis.

In addition, we showed that ATRA significantly reduces the viability of BTZ-treated cells, proving that Nrf2 is crucial in inducing cell resistance. These results are confirmed by over-expressing a dominant negative form of Nrf2, in order to inhibit the endogenous protein. The viability of Nrf2-DN cells is significantly reduced by BTZ treatment. However, Nrf2 inhibition, obtained by using both ATRA or Nrf2-DN, is not able to reach the efficacy of the association of BSO and HO-1 silencing in improving BTZ efficacy. In fact, ATRA treatment is not able to completely prevent the binding of Nrf2 to the promoter regions of its target genes and Nrf2-DN does not completely prevent Nrf2-dependent gene induction in BTZ-treated cells. Therefore, we can hypothesize that a more efficient inhibition of Nrf2-ARE binding could exert better results in reducing chemoresistance.

Hence, these data highlight the key importance of Nrf2, HO-1 and GSH as molecular targets in order to sensitize high-risk neuroblastoma to proteasome inhibition obtained by using low doses of bortezomib, so improving the chance of an efficient therapeutic application of bortezomib as a sensitizing chemotherapeutic agent with low side-effects.

## Supporting Information

S1 Fig siHO-1 reduces BTZ-induced HO-1 upregulation. siHO-1 was performed as detailed in Materials and Methods section. Increasing concentrations (10–50 nM) of siHO-1 have been used in order to set up the best experimental condition and the dose of 50 nM has been used for the following experiments. No changes were observed using non-targeting siRNA or Interferin alone (data not shown). The bands (a) show one representative experiment and the graph (b) shows the mean value of three independent experiments (mean±SE); *p<0.05 vs BTZ-treated cells.
(PDF)

S2 Fig BSO strongly reduces total GSH content in HTLA-230 treated with BTZ and siHO-1. tGSH amount was measured by mean of HPLC analysis as described in Materials and Methods section. HTLA-230 cells were treated as indicated. The graph shows the mean value of three independent experiments (mean±SE); n.d. = not detectable; *p<0.05 vs untreated cells.
(PDF)

S3 Fig Nrf2-DN over-expression reduces BTZ-induced HO-1, GCLM and x-CT up-regulation. mRNA and protein levels of HO-1 (a and d, respectively), GCLM (b and e, respectively) and x-CT (c and f, respectively) were measured by means of RT-PCR and Western Blotting in cells transfected with a plasmid coding for a dominant negative form of Nrf2 (pmax-Nrf2-DN) or an empty plasmid (pmax-empty vector) as an internal control and then treated with 2.5 nM BTZ. The graphs show the mean value of three independent experiments (mean±SE). The bands show one representative experiment. #p<0.05 vs untreated cells; *p<0.05 vs BTZ-treated cells.
(PDF)

## Abbreviations

BTZ: bortezomib
Nrf2: nuclear factor-erythroid-derived 2-like 2
HO-1: heme oxygenase 1
GCLM: γ-glutamylcysteine ligase, light subunit
GCLC: γ-glutamylcysteine ligase, heavy subunit
x-CT: cysteine/glutamate transporter
ARE: antioxidant responsive element
ATRA: all-*trans*-Retinoic acid
NB: neuroblastom
GSH: glutathione
TED: TAT-EDANS-DABCYL
BSO: buthionine sulfoximine
Nrf2-DN: Nrf2 dominant negative
