# Supplementary figures and images for: Role of Nrf2, HO-1 and GSH in Neuroblastoma Cell Resistance to Bortezomib
Source: PLoS One. 2016 Mar 29;11(3):e0152465. doi: 10.1371/journal.pone.0152465 (PMC4811586; doi:10.1371/journal.pone.0152465)

S1

a

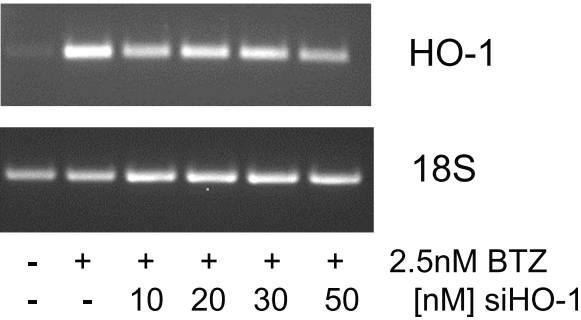

b

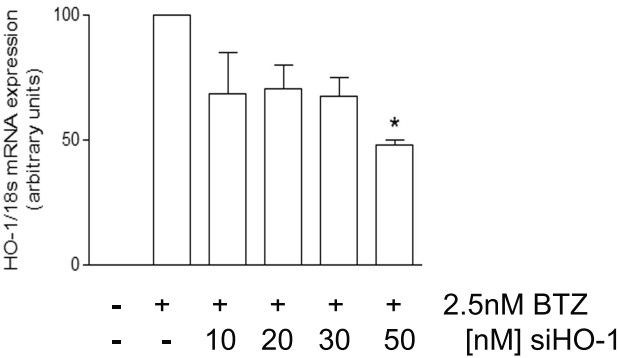

Supplement: S1 Fig — siHO-1 was performed as detailed in Materials and Methods section. Increasing concentrations (10–50 nM) of siHO-1 have been used in order to set up the best experimental condition and the dose of 50 nM has been used for the following experiments. No changes were observed using non-targeting siRNA or Interferin alone (data not shown). The bands (a) show one representative experiment and the graph (b) shows the mean value of three independent experiments (mean±SE); *p<0.05 vs BTZ-treated cells. (PDF) [file pone.0152465.s001.pdf]

S2

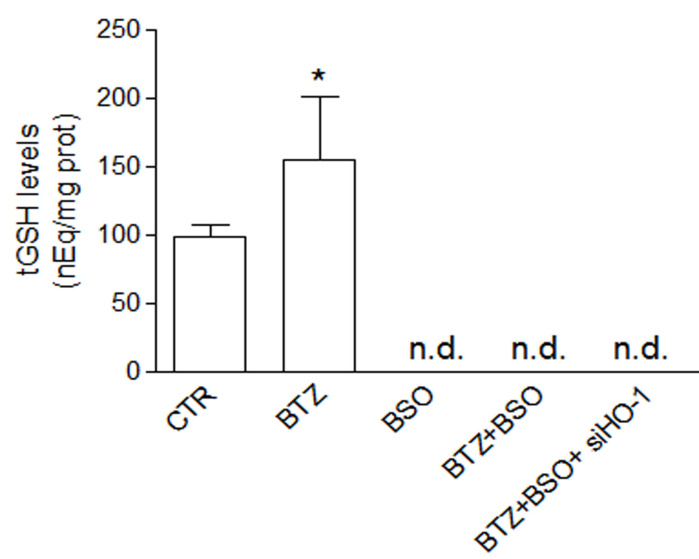

Supplement: S2 Fig — tGSH amount was measured by mean of HPLC analysis as described in Materials and Methods section. HTLA-230 cells were treated as indicated. The graph shows the mean value of three independent experiments (mean±SE); n.d. = not detectable; *p<0.05 vs untreated cells. (PDF) [file pone.0152465.s002.pdf]

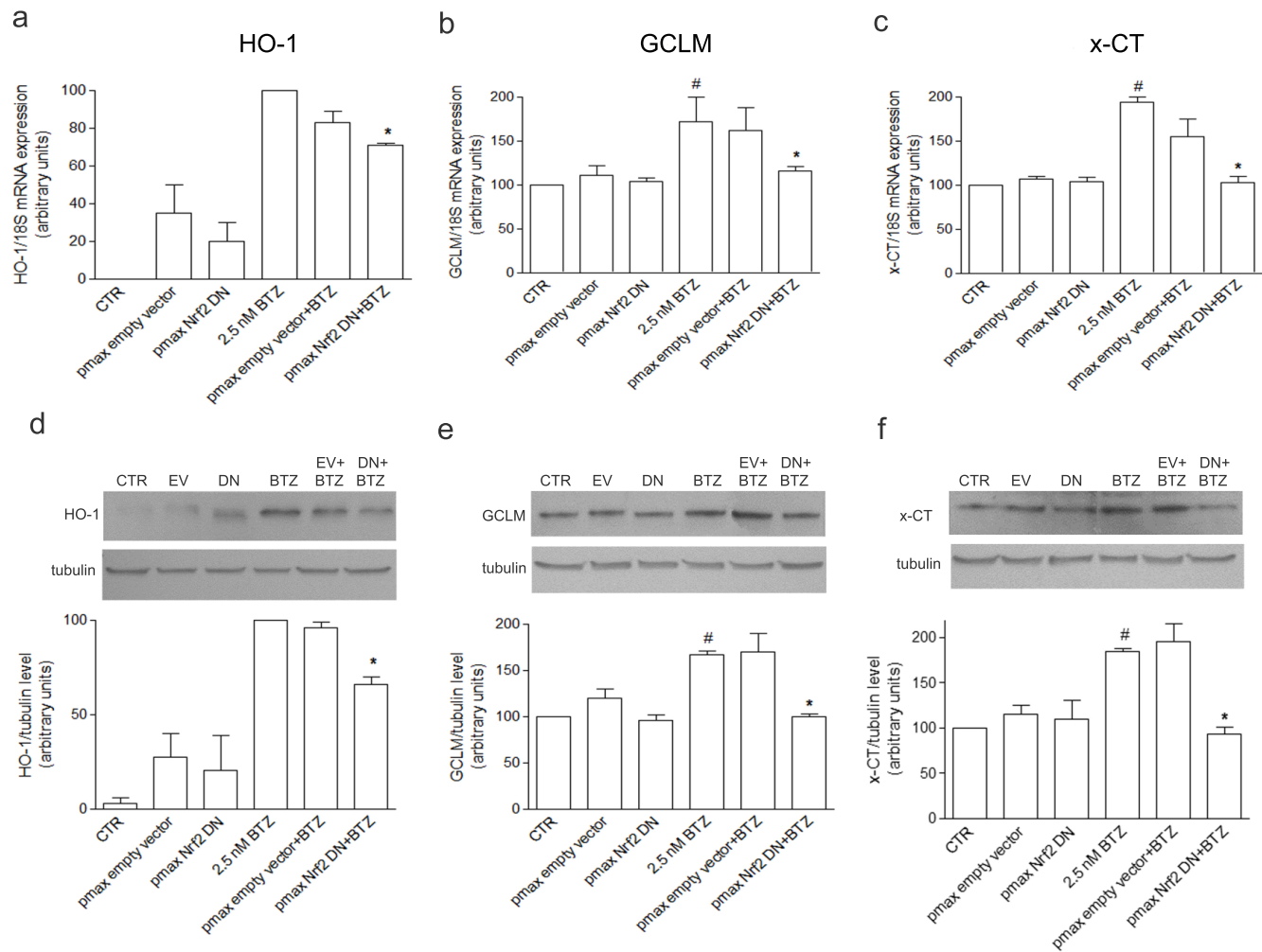

Supplement: S3 Fig — mRNA and protein levels of HO-1 (a and d, respectively), GCLM (b and e, respectively) and x-CT (c and f, respectively) were measured by means of RT-PCR and Western Blotting in cells transfected with a plasmid coding for a dominant negative form of Nrf2 (pmax-Nrf2-DN) or an empty plasmid (pmax-empty vector) as an internal control and then treated with 2.5 nM BTZ. The graphs show the mean value of three independent experiments (mean±SE). The bands show one representative experiment. #p<0.05 vs untreated cells; *p<0.05 vs BTZ-treated cells. (PDF) [file pone.0152465.s003.pdf]
